# Supplementary figures and images for: Training load quantification of high intensity exercises: Discrepancies between original and alternative methods
Source: PLoS One. 2020 Aug 3;15(8):e0237027. doi: 10.1371/journal.pone.0237027 (PMC7398532; doi:10.1371/journal.pone.0237027)

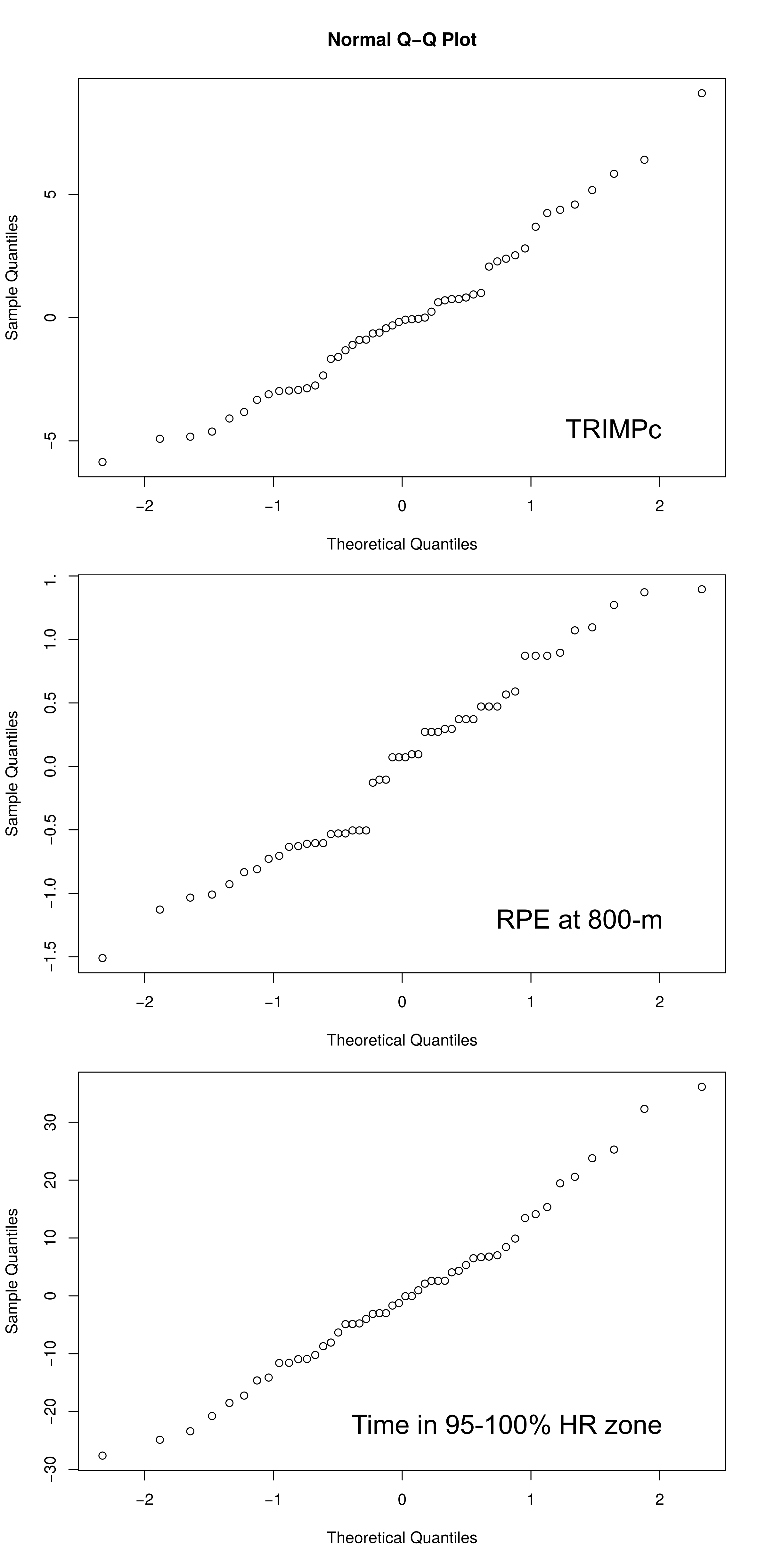

Supplement: S1 Fig — (TIF) [file pone.0237027.s001.tif]

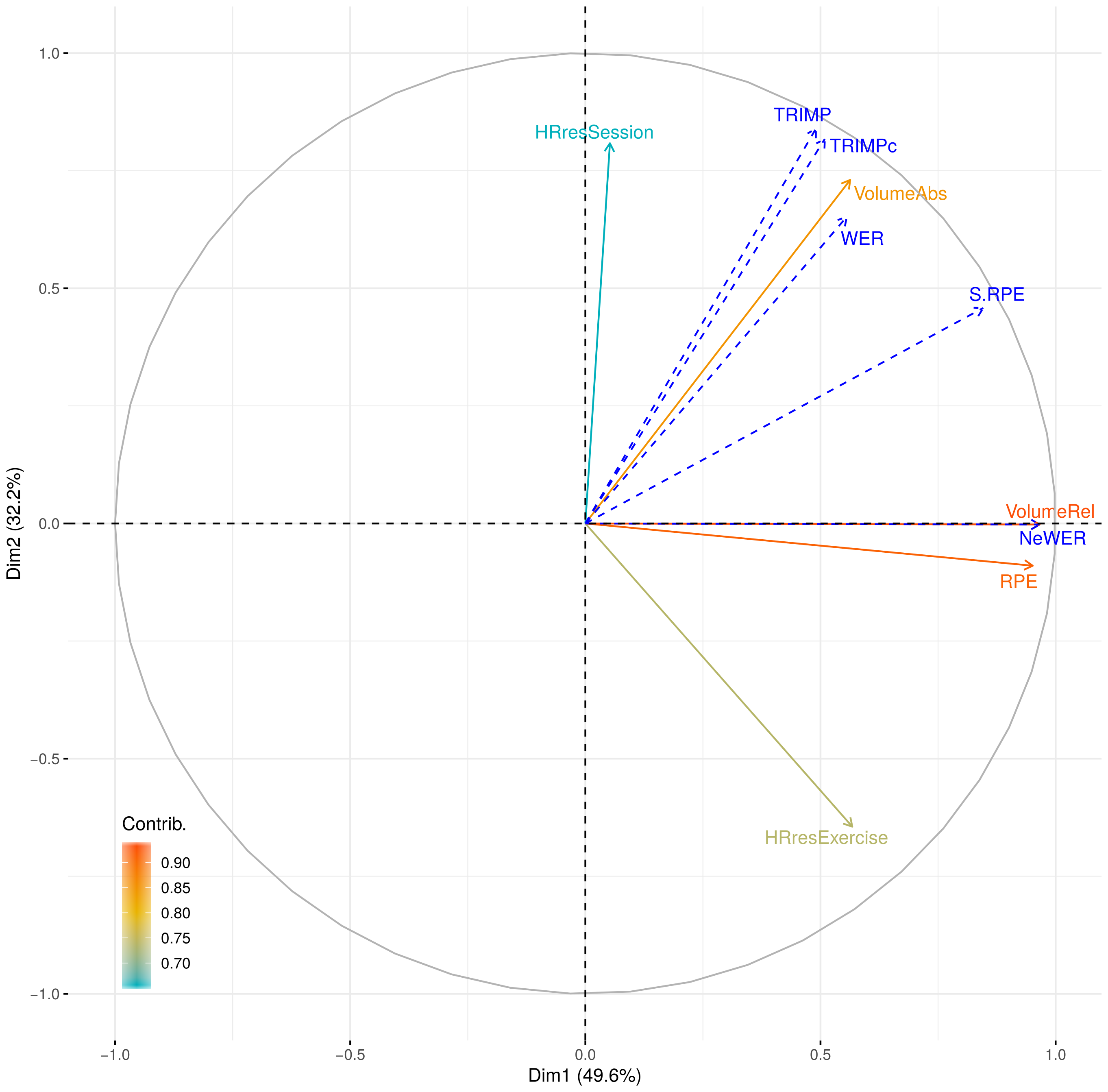

Supplement: S2 Fig — (TIF) [file pone.0237027.s002.tif]
