# Supplementary material for: Training load quantification of high intensity exercises: Discrepancies between original and alternative methods
Source: PLoS One. 2020 Aug 3;15(8):e0237027. doi: 10.1371/journal.pone.0237027 (PMC7398532; doi:10.1371/journal.pone.0237027)
Supplement: S1 Appendix — (DOCX) [file pone.0237027.s003.docx]

S1 Appendix

We used two complementary approaches to determine the link between the measured (RPE, absolute volume, relative volume, session’s HR and exerceise HR) and the calculated (session-RPE, WER, NeWER, TRIMP and TRIMP-c) variables. The first one was a mixed linear regression model to determine for each of TL the R^2^ contribution averaged over orderings among regressors with lme4 package (measured variables). The second was a Principal Analysis Component (FactoMineR package) to visualize the relative contribution of each of the original variables, calculated TL were used as supplementary variables to confront them to the principal components.
