# Supplementary material for: Training load quantification of high intensity exercises: Discrepancies between original and alternative methods
Source: PLoS One. 2020 Aug 3;15(8):e0237027. doi: 10.1371/journal.pone.0237027 (PMC7398532; doi:10.1371/journal.pone.0237027)
Supplement: S2 Appendix — (DOCX) [file pone.0237027.s004.docx]

## S2 Appendix

Linear regression models describe that absolute volume was the main contributor to TL in WER, S-RPE, TRIMP and TRIMP_c_ methods (respectively, R^2^=0.61, 0.38, 0.70 and 0.74) and relative volumes to RPE_alone_ (R^2^=0.67). As NeWER and relative volume were strongly correlated R2=1, we excluded the relative volume from the measured parameters in the linear regression model and the main contributor to NeWER was RPE_alone_ (R^2^=0.66).
