# Supplementary material for: Training load quantification of high intensity exercises: Discrepancies between original and alternative methods
Source: PLoS One. 2020 Aug 3;15(8):e0237027. doi: 10.1371/journal.pone.0237027 (PMC7398532; doi:10.1371/journal.pone.0237027)
Supplement: Attachment — Submitted filename: Responses to reviewers comments.docx [file pone.0237027.s005.docx]

**Training load quantification of high intensity exercises: discrepancies between original and alternative methods**

**Responses to reviewers’ comments.**

Many thanks for comments that help us to markedly improve our article. Responses appear in italics after the comment and leading to changes in the manuscript these appear underlined in the text. F Desgorces, P Noirez

Reviewer #1: The manuscript titled, “Training load quantification of high intensity exercises: discrepancies between classical and alternative methods,” presented a research study with the main aim of comparing classical and alternative methods of quantifying training load during high intensity exercises. The mode of exercise included five sessions of outdoor running on a track: 1) 800-m maximal effort; 2) 400-m intervals until exhaustion with a 2:1 work to recovery ratio; 3) 400-m intervals until exhaustion with a 1:1 work to recovery ratio; 4) 400-m intervals until exhaustion with a 1:2 work to recovery ratio; and 5) 200-m intervals until exhaustion with a 1:1 work to recovery ratio. The topic is of practical importance and can help guide athletes, coaches, and sport scientists on the best methods to quantify training load for high intensity sessions such as track interval work. The authors main findings were that the training load calculations are not interchangeable. Their recommendation is that when the exercise session involves exhaustion, alternative training load calculations should be used that account for exhaustion and perceived exertion in their calculations.

Major Comments:

1) The manuscript includes a number of grammatical errors. I would suggest the authors address this to the best of their ability. This will improve clarity as well as sentence structure.

*We conducted a careful and complete proofreading of the paper (changes are not all underlined in the text).*

2) In your discussion of training load, it would be helpful to identify and define internal and external training load. The dose of exercise is the external training load (800-m sprint for example). The response to the dose of exercise is the internal training load (RPE for example).

*It is voluntary to not use the terms of internal/external training load as these terms lead to our point of view in misunderstanding of what is training load, then such a use should require a complete explanation that can be too long and not the point of our article. Training load is defined as the dose of exercise according to the physiological strain induced. Training load quantification is the assessment of the exercise dose that could be used to analyze and understand the exercise effects. In the literature we can identify quantification methods called internal methods as they are based on physiological parameters to assess the exercise intensity (HR or blood lactates) or bio-psychological parameters (RPE), we assume that WER or NeWER based on individual exhaustion are methods for internal TL quantification. Internal TL cannot be the response to the dose as the aim is to use them to analyze the response to the dose; HR, blood lactate, RPE are not used by TL quantification methods as response to the dose but as parameters describing the exercise intensity (perhaps also as describing density). To our point of view, external TL could be the dose that is not expressed relative to individual capacities. Nevertheless, we are not aware of an existing method that provide external TL, perhaps Total load from GPS are external TL as speeds or acceleration data are not expressed relative to individual capacities. In our point of view, 800m sprint is not a TL but a work load, an exercise volume in an intensity zone. The difference being that such volume cannot be accumulate to describe training programs; how 800m at sprint velocity can be added to 20km at moderate intensity without minimizing the 800m sprint (although 800m may be very hard) ?*

3) There is not much literature on the work endurance recovery formula. The authors should justify why they believe this is a classical method. Please provide more background on where it came from and reasoning for creating an alternative formula to the work endurance recovery formula. My understanding from reading reference #3 is that the calculation provides a more accurate training load quantification when a training session included intermittent exercise such as a strength training session.

*We replace the term of classical by original throughout the text (including in the title of the article). We add some description of the WER method and of papers using it (lines 88-96).*

4) It is unclear whether the work to recovery ratio was time or distance. For example, in the 400m 2:1 ratio, did the participants run the 400-m interval and then walk-jog for 200m recovery or did they have a rest period of half the duration of the 400-m interval? Please clarify this.

*Added “(based on a duration index) line 145-146.*

5) The authors defined TL800 as a distance of 800-m at specific velocity. It is unclear what portion of the workout this 800-m is taken from. From Table 1 it appears some of the 800-m segment includes recovery distances. I believe it would be more relevant to compare 800-m from each workout at the 800-m race velocity.

*Through this and next comments we understand that the “exercise total time” of table 1 was confusing. This “exercise” total time was only informative and not used in quantification methods, we decided to replace “exercise total time” by “session total duration” which is used by quantification methods (warm-up and cool-down durations added to exercise time).*

*The exercise total duration included recovery durations which differed according to sessions; if recovery was not included, whatever the session, 800-m runs at the same velocity required the same time.*

*Modification of line 159 “…*from the warm-up until the completion of 800m of run at the 800m performance velocity. *”*

6) Is it the authors’ position that training load for each workout should be similar because each workout achieved maximal physiological strain (done to exhaustion)? I believe the benefit of rest periods in interval sessions is that the athlete can perform more work overall. Thus the training load for the 400m 1:2 and 200m 1:1 should be higher. The athlete was able to run farther at 800-m velocity. This additional work should be taken into account. Stephen Seiler’s work compares a fixed amount of work across a range of interval prescriptions. This is what I thought TL800 was meant to be, but the data indicates TL800 includes recovery periods.

*As stated above, S-RPE and TRIMP methods are based on session duration that includes recovery pauses that result in highest TL800 for the easiest sessions (400m 1:2 and 200m 1:1, easiest when only 800m were accumulated).*

*We do not know all the works of S Seiler but, to our knowledge, he mainly works with exercise volume expressed in given intensity ranges so with workloads rather than TL. The aim of TL is to quantify the doses of exercise in an unique value. It may be difficult to accumulate sprint and endurance volumes without under-expressing sprint (intensity influence was then ignored). TL is a combination of exercise volume-intensity-density, these 3 parameters have to be accounted together equately. In the present study, easiest work-recovery ratio (1:2) and interval distance (200m) induced physiological conditions that allow to run farther. The additional work corresponds to volume increase that occurs with decreased interval distance and work-recovery ratio. In the 800m performance, total volume was lower but interval distance and work-recovery the highest.*

*Our position is that, if TL corresponds to the exercise dose according to exercise’s intensity-volume-density and to the physiological strain induced, the highest physiological strain should result in the highest TL. If exhaustion is reached in a session that suggests that the maximal physiological strain was achieved, no higher strain considering the session construct is possible. This highest strain should correspond to the highest TL.*

7) I believe if the authors presented the findings in a more unbiased way, it would strengthen the paper. In addition, the paper would be greatly strengthened if the paper had more clarity regarding methodology. A figure showing the segment of TL800 would help.

*We assume that our point of view may be bewildering; we edit some sentences of our paper to reduce the impression of biased presentation.*

*A figure 1 was added, it also describes TL total and TL800 limits.*

I will reserve a majority of my minor comments with the revision. However a few are listed below :

1) It looks neater when the formulas are separated out from the main text.

*As suggested by authors guidelines, formulas have been inserted by using Microsoft equation insert.*

2) Please indicate how you defined exhaustion (when participants stopped the interval session).

*Added line 139-140 “*Here exhaustion designates the inability of a subject to maintain the expected velocity during two 100m intervals in a row*”*

3) Please clarify what you mean by density of exercise.

*Inserted in the introduction line 61-63 “…iii) density caused by recovery period duration (frequently expressed through work: recovery ratio) and sometimes by the intensity of the recovery time…”*

4) Did the researchers record temperature, wind speed, and/or humidity for the training sessions? Please indicate.

*Air temperature and hygrometry added LINE 124-125. Wind conditions have not been precisely measured in the track location.*

Reviewer #2: The paper " Training load quantification of high intensity exercises: discrepancies between classical and alternative methods" is very interesting. This study is in a relevant area and be should be replied with other type of participants or sports. The authors should be commended for their hard work in what appears to be an extensive study. In my point of view, the current form of the manuscript does not provide practical impact in research field, but it may provide if some further details were given. Hence, I've recommended revision to improve further text clarity before I can consider recommending it for publishing.


Abstract
L 27-29 – I believe that would be better if you simplify the sentence to “The purpose of the present study was to quantify training loads (TL) by classical methods (TRIMP; session-RPE; work-endurance-recovery) and their alternatives (TRIMPcumulative; RPEalone; New WER).”

*Sentence changed.*

L 29 – The author should avoid beginning sentences with numbers.

*10 replaced by “Ten”.*

L 45-46 – I don’t really understand what you mean. You should focus on presenting your conclusions.

*We added some results in the abstract Line 35-37. The end of the conclusion was changed.*

L46-48 – Although I understand that authors want to make some suggestions for future research, I would prefer to read some main conclusions in the last lines of the abstract. Otherwise, a reader will keep the idea that.

*See previous response.*

Manuscript
L52 – It should be “forty-five years ago”.

*Yes! change done.*

L67-68 – There a lot of scientific papers that says the opposite. I would be careful with sentences like this.

*Sentence slightly changed to “…some authors suggested that no method”.*

L171-172 – I not sure If I understand what you mean. You assumed that HRmax was the highest HR reached during 800-m running? If so, I do not agree with this procedure. Did you consider accessing HRmax by applicating a maximum cardiorespiratory test? How can you confirm that HRmax achieved in the 800-m running was the HRmax that a participant can achieve?

*Not really, the highest HR reached during all the sessions was considered as HR max, but in fact, the highest HR was recorded during the 800-m performance. By experience in middle distance and rowing, HR reached during cardiorespiratory tests may be lower than during a maximal exercise (of mid duration). Similar or higher HR or VO2max in performance than in tests were reported by some studies (e.g., Thomas et al. Oxygen Uptake Response to an 800-m Running Race. Int J Sports Med, 2004, 26, 268-273.).*

L183-186 – you recorded RPE 30min following the end of the session, but you also recorded immediately after the 800-m performance. How do you use both values? I suggest a better clarification.

*This part (line 209-213) has been improved. For its use in S-RPE quantification method, RPE has to be recorded 30min following the end of the session. That was what we did for TLtotal quantification. To quantify TL800, because sessions were not finished, we recorded RPE immediately when 800m of run were accumulated in intermittent sessions while sessions were not terminated, and immediately after the 800-m performance (for TL800 although 30 min after the cool-down RPE was re-assessed).*

*The figure added as suggested by reviewer 1 would help the reader to understand this point.*

L225 – it is not clear what does mean letters a, b, c, d and e. they are significantly different from…?

*We choose this presentation because so many signs would be necessary to indicate specific differences. Therefore, two results with a similar superscript letter are not different, different superscript letters describes differences between the data.*

L239-240 and 247-253 – The same comment regarding figures 1 and 2.
Also, it is recommended to include SD in the figures presented.

*Added in the legends. “Data are presented as mean ± SD.”*

L277-278 – This is speculative. Can you point what were the previous analysis that you are referring?

*This sentence has been removed of the text and replaced by “For accurate TL quantification, causes of the differences between classical and alternative methods have to be clarified.”*

L302 – what do you mean by achieving exhaustion? Is that mean that a participant can no longer maintain their exercise intensity?

*Added line 139-140 “*Here exhaustion designates the inability of a subject to maintain the expected velocity during two 100m intervals in a row*”*

L303-304 – I want to ask if the authors can provide the analysis with including warm-up and cool down session. Despite the majority of the studies regarding TL quantification include those parts of the session, it would be interesting to analyse the differences between the methods used without including warm-up and cool down.

*Interested suggestion (we understand excluding warm-up and cool-down), unfortunately for a complete work we don’t have RPE at the end of warm-up and immediately at the end of runs (without cool-down). It is possible to calculate WER and NeWER without warm-up and cool-down that will result in very light TL changes. If we do that with TRIMP, TL from warm-up (20min) and cool-down (10min) will be higher than those from 800m performance and 400m 2:1. In S-RPE method, we can hypothesized that warm-up induced a RPE of 1-2 for 20min would led to a TL of 20-40 ua, while the 800m performance of 2min with RPE of 10 would led to 20 ua.*

L322-323 – That assumption seems obvious. In order to clarify, can you provide some guidelines for future use or how to choose a method to TL quantification? Can you add in Practical applications section?

*The sentence has been removed of the text, and a practical applications and limitations section has been added.*

L324-333 – I don’t know how that paragraph contributes with new knowledge. There are a lot of studies that analysed the construct and validity of RPE or S-RPE that already state what you mention. I know you are trying to build a text with logical following, but can you add any new advice regarding this topic?

*This part –originally line 324-345- has been replaced just before the conclusion and was used to build a new section for “practical applications and limitations”.*

L339 – “…may be uneasy and inefficient for determining TL of exercises or programs to come”. Can you clarify what you mean? Because I can enumerate a lot of studies that support the use of RPE.

*We agree the comment. This part (line 373-383) was changed for a better understanding notably for future applications.*

L352 – I think there are something missing in the end of the sentence, once you put “()” with no information on it.

*Removed of the text.*

L372 – I suggest that you specify some methods by giving some examples.
In addition, there no Practical applications or limitations presented. Those sections should be reviewed. For instance, after reading, a reader still does not know what TL quantification method should use. Also, some limitations, e.g. the small sample size should be noticed.

*A “practical application and limitation” part has been added at the end of our text just before the conclusion.*

Reviewer #3: 1. Is the manuscript technically sound, and do the data support the conclusions?
The purpose of the study is of interest in the field of training and athletic preparation. Unfortunately, to my opinion, the cohort is very small and do not allow generalization of the results obtained, especially based on the statistical procedure used. Authors should state this at the end of the discussion section. The protocol is well conducted and supports the replication of the study.

2. Has the statistical analysis been performed appropriately and rigorously?
To my opinion, the statistical analysis which has been performed is the main weakness of the study. Based on 10 athletes (which is a very small cohort), the authors tested the normality of the data using a Kolmogorov-Smirnov test. According to the state of my knowledge, for small samples, it is currently recommended to use the Shapiro–Wilk test. Indeed, a test of significance for normality may lack power to detect the deviation of the variable from normality. Shapiro–Wilk test was designed to test for normality for small data-size (n < 50). This test is more powerful than Lillifors or Kolmogorov-Smirnov and other tests for small data-size. Then I recommend making a Shapiro–Wilk test to assess the normality of the data.

*In fact RPE results and associated TL appeared heterogeneous and not normally distributed. We change the statistical part and method (line 215-226). Variance homogeneity was tested by using Levene test and data normality was assessed graphically (examples in S1).*

Based on the Kolmogorov-Smirnov test, authors have made one-way ANOVA and Pearson’s correlations. Using ANOVA needs that the residuals respect the normality assumption. In this line, and considering the small number of subjects, I recommend putting the QQ-plot in the supplementary materials so that others can see what authors judged to be normal, regardless of what their normality test says about residuals. I think it is important because such things are tricky with small sample sizes.

*As stated above statistical analysis part has been changed, ANOVA was used for homogeneous variances whereas Spearman test was used for heterogeneous variances. Examples of QQ plots were added in S1.*

Another matter considering the results obtained in this study is the power of the tests. In this line, I recommend two things. Firstly, please provide power analysis to estimate if the sample size is large enough for the protocol and statistical analysis used. Secondly, add in the results section effect size and/or confident intervals (ANOVA, correlations) to support the results and to allow discussions.

*Effect sizes were added in table 1 and correlations confidence intervals in table 2.*

3. Have the authors made all data underlying the findings in their manuscript fully available?

Authors report that some restrictions will apply and that all relevant data are within the manuscript and its Supporting Information files.

4. Is the manuscript presented in an intelligible fashion and written in standard English?
Line 55. TL is defined as the exercise-induced physiological strain resulting from the combination of exercise’s intensity, volume and density influences.
Please add a rationale for using the RPE scale which includes a subjective aspect in the training load evaluation.

*A rational of RPE use has been added in the text (Line 79-82).*

Line 65. As example, pharmaceutics weight is linked to their weighing and not to their expected effects. Please, provide a clearer example, or perhaps elaborate on the argument.

*This sentence has been removed of the text.*

*Added in the text lines 67-71. “…*However, some authors consider that exercises with higher volumes should result in higher TL based on their expected major effect on performance. Unfortunately, this approach leads to predefining some of the exercises’ TL prior to the analysis of the dose-response relationship. We posit that TL should remain a measure of the exercise dose without considering its expected effects*.”*

L 100. They were in good mental and physical condition, and were not under any medication.
Please, provide information concerning how these aspects were controlled.
*Sentence slightly changed (line 113-115).*

L 109. Each session was performed at 10.00 a.m. on an outdoor athletics track with similar climatic conditions. Subjects were asked to arrive for each session in a rested state, suspend their normal training for at least 24h prior to session and avoid exhausting exercises in the study period.

This means changing the usual athletes’ training program. In your opinion, what would have been the consequences of the prior charges on the states? Moreover, what is the point of doing that since we are going to try to measure a load induced by specific exercises? Shouldn't the effect you observed be independent of the initial form state? The fitness standardization is commendable but wouldn't it have been better to have a population carrying out the same training program during the 3 weeks of the study? Please, give a rationale for this standardization.

*We agree the comment, studies have to balance between usual training and protocol unbiased by accumulated fatigue. Our subjects were usually trained from 4 to 6 days/week and the protocol imposed 5 days off in three weeks that might be more that usually for some subjects but usual for others. However, if our protocol was based on usual exercise types for our subjects the fact that each session was pushed until exhaustion actually induced fatigue. Five sessions to exhaustion in three weeks is unusual for our subjects (and many others), so we assume to avoid other exhausted sessions. Added in the text (lines 126-127) “*The study protocol increased the number of exhausting sessions at high intensity, thus changing subjects’ training habits. Subjects were asked …*”*

L 121. interspersed by at least three days. Please, give Mean/SD.

*Added line 138. “interspersed by at least three days (3.5 ± 0.6 days).”*

L 172. HRb was self-measured when the subjects awoke in three consecutive mornings, the lower value measured was recorded. Please, provide information on the process that has been followed. Anatomical position and recording duration.

*Added line 198-199. “HR_b_ was self-measured in the supine position during a 3-min period when the subjects awoke in three consecutive mornings”*

L 179. RPE was obtained using the category ratio scale (CR-10) modified by [15].
1. Please argue why you used this tool instead of the original one.

*To our knowledge, CR-10 scale modified was adapted by Foster et al for being suitable in ratings during and following exercise training sessions (vs exercise tests). Obviously, the S-RPE method was developed, and used by hundreds of papers with this modified CR-10 scale, we used the method as suggested by their authors.*

2. Rewrite [15]

*Done*
L 330. Therefore, RPE may reflect exercise intensity but also relative volume and density, suggesting that, in S-RPE method, exercise volume is accounted twice (i.e., in RPE and in volume itself). Conversely, RPE itself could be used to monitor and assess, at an individual basis, the exercise-induced physiological strain that is the definition of TL.

To my knowledge, the session-duration refers to the length of session expressed in minutes while the nominal score is given by an athlete to describe his RPE of “mean training intensity” during that training session. In this perspective, there is no reason to delete the time volume from the calculation. Please add a more consistent rationale supporting this sentence.

*Comment of reviewer 2 “*There are a lot of studies that analyzed the construct and validity of RPE or S-RPE that already state what you mention.*”*

*We slightly change this part.*

*We agree we this comment, S-RPE method was built by multiplying a volume parameter (duration) by an intensity parameter (RPE). However, in exercise or training session, when you ask to subjects “How much exertion are you feeling right now” the exercise duration influences the rating. If you ask the question after 10min of run at 12 km/h and after 1h at the same speed, the rating will change. That was not due to the scale used in S-RPE method but demonstrated in numerous other studies, exertion is not solely influenced by intensity but by all parameters of exercise.*

*We add some sentence to strengthen this point. Line 322 “*The higher interval distances and work:recovery ratios the higher the RPE.*”*

*Line 377-380.”* In our results, RPE was markedly increased by the accumulation of runs (RPE at 800m *vs* at sessions’ end) and by increased work:recovery ratios as observed after 800m accumulated runs. This demonstrates the impact of exercise’s duration and density on ratings, supporting the use of RPE_alone_ to quantify the exercise dose.*”*

L 352. than exercise intensity or density ().

Please, delete brackets or add items.

*Deleted*
